# Analysis of Guanine Oxidation Products in Double-Stranded DNA and Proposed Guanine Oxidation Pathways in Single-Stranded, Double-Stranded or Quadruplex DNA

**DOI:** 10.3390/biom4010140

**Published:** 2014-02-10

**Authors:** Masayuki Morikawa, Katsuhito Kino, Takanori Oyoshi, Masayo Suzuki, Takanobu Kobayashi, Hiroshi Miyazawa

**Affiliations:** 1 Kagawa School of Pharmaceutical Sciences, Tokushima Bunri University, 1314-1 Shido, Sanuki, Kagawa 769-2193, Japan; E-Mails: s110702@stu.bunri-u.ac.jp (M.M.); s120002@stu.bunri-u.ac.jp (M.S.); kobayashit@kph.bunri-u.ac.jp (T.K.); miyazawah@kph.bunri-u.ac.jp (H.M.); 2 Faculty of Science, Department of Chemistry, Shizuoka University, 836 Ohya, Suruga, Shizuoka 422-8529, Japan; E-Mail: stohyos@ipc.shizuoka.ac.jp

**Keywords:** DNA damage, electron transfer, photooxidation, 8-oxo-7,8-dihydroguanine

## Abstract

Guanine is the most easily oxidized among the four DNA bases, and some guanine-rich sequences can form quadruplex structures. In a previous study using 6-mer DNA d(TGGGGT), which is the shortest oligomer capable of forming quadruplex structures, we demonstrated that guanine oxidation products of quadruplex DNA differ from those of single-stranded DNA. Therefore, the hotooxidation products of double-stranded DNA (dsDNA) may also differ from that of quadruplex or single-stranded DNA, with the difference likely explaining the influence of DNA structures on guanine oxidation pathways. In this study, the guanine oxidation products of the dsDNA d(TGGGGT)/d(ACCCCA) were analyzed using HPLC and electrospray ionization-mass spectrometry (ESI-MS). As a result, the oxidation products in this dsDNA were identified as 2,5-diamino-4*H*-imidazol-4-one (Iz), 8-oxo-7,8-dihydroguanine (8oxoG), dehydroguanidinohydantoin (Ghox), and guanidinohydantoin (Gh). The major oxidation products in dsDNA were consistent with a combination of each major oxidation product observed in single-stranded and quadruplex DNA. We previously reported that the kinds of the oxidation products in single-stranded or quadruplex DNA depend on the ease of deprotonation of the guanine radical cation (G^•+^) at the N1 proton. Similarly, this mechanism was also involved in dsDNA. Deprotonation in dsDNA is easier than in quadruplex DNA and more difficult in single-stranded DNA, which can explain the formation of the four oxidation products in dsDNA.

## 1. Introduction

**Figure 1:**
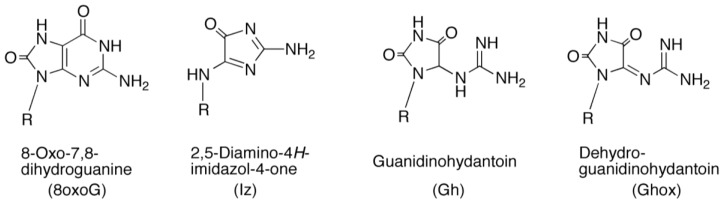
Structures of guanine photooxidation products.

Environmental agents, such as UV-light or free radicals, often oxidize DNA bases and oxidation of DNA bases is accepted as one of the principal sources of genetic damage involved in genetic mutation, aging, and cell death [1,2,3,4,5,6,7,8]. Since the oxidation potentials for guanine, adenine, cytosine, and thymine are 1.29, 1.42, 1.6, 1.7 V, respectively [9], guanine is the most easily oxidized among the DNA bases and can form several oxidation products (Figure 1). Guanine-rich sequences exist in many important genomic regions, such as telomeres [10,11,12,13] and the promoter element of the proto-oncogene c-myc [14,15], and these sequences can fold into quadruplex structures in the presence of suitable metal ions. Previously, the oxidation products of quadruplex DNA were compared with that of single-stranded DNA in a study using 6-mer DNA d(TGGGGT), which is the shortest oligomer among the quadruplex-forming sequences in the presence of several ions [16]. As a result, the photooxidation products of quadruplex DNA were found to be significantly different from that of single-stranded DNA [17]. In quadruplex DNA, the main oxidation products formed are 8-oxo-7,8-dihydroguanine (8oxoG) and its oxidation product, dehydroguanidinohydantoin (Ghox), whereas the main product formed in single-stranded DNA is 2,5-diamino-4*H*-imidazol-4-one (Iz). The discussion that DNA structure influences the types of photooxidation products is important, and similar discussions have been reported [18,19]. Additionally, the spiroiminodihydantoin-2’-deoxyribonucleoside, which was formed by one-electron oxidation in a previous study [20], was not detected in our study [17].

The types of guanine oxidation products depend on the structure of quadruplex or single-stranded DNA [17]. Therefore, we thought that the photooxidation products of double-stranded DNA (dsDNA) may differ from that of quadruplex or single-stranded DNA, and that the differences in these guanine oxidation products are likely to explain the influence of DNA structures on guanine oxidation pathways. To fully understand differences in guanine oxidation pathways in single-stranded, double-stranded and quadruplex DNA, we attempted a direct, simultaneous analysis of the types, yields, and sites of photooxidation products in dsDNA. Using d(TGGGGT) [17], the oxidation products in dsDNA were compared with the products in single-stranded or quadruplex DNA.

## 2. Results and Discussion

### 2.1. Formation of Double-Stranded DNA

**Figure 2:**
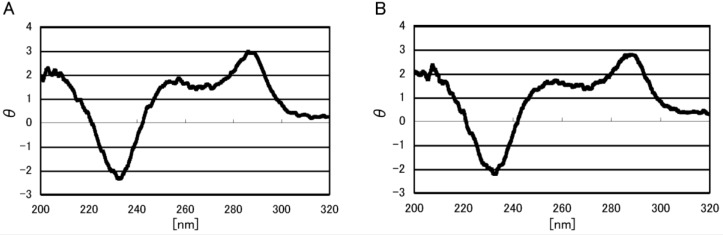
The effect of RF on the formation of double-stranded DNA. CD spectra obtained with 70 µM d(TGGGGT) and 70 µM d(ACCCCA) in 10 mM KCl, 5 mM cacodylate buffer (pH 7). CD spectroscopy was performed (**A**) without RF or (**B**) with 7.5 µM RF.

The double-stranded structure of the 6-mer oligomers was formed in a KCl solution. d(TGGGGT) and d(ACCCCA) (70 µM each) in 10 mM KCl were heated to 80 °C for 5 min, incubated at 4 °C for 1 min, and the DNA structure was determined using circular dichroism (CD) spectroscopy. Since an increase in the CD spectrum around 250–300 nm, which is typical of B-form dsDNA, was detected in 10 mM KCl (Figure 2A) [21], the formation of dsDNA was confirmed. Additionally, the predicted melting temperature [22] also supports the formation of dsDNA.

**Figure 3:**
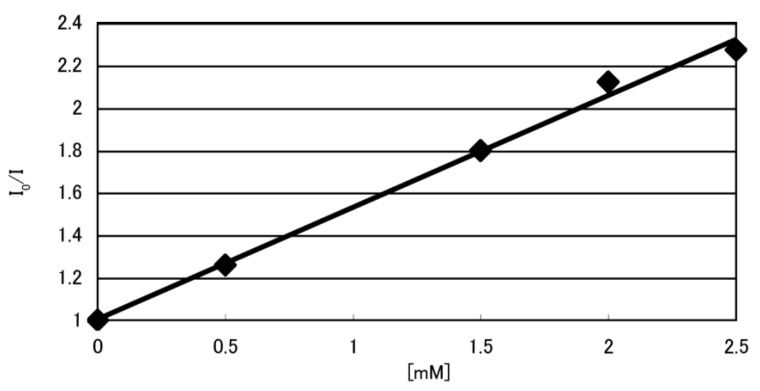
Fluorescence quenching of RF. Fluorescence quenching of 6.25 µM RF by double-stranded DNA (0–2.5 mM) in 10 mM KCl, 5 mM cacodylate buffer (pH 7) was performed. I_0_/I indicates the ratio of fluorescence intensities.

In this study, dsDNA was oxidized using UVA radiation with riboflavin (RF, *i.e.*, vitamin B_2_). To determine the binding of RF to DNA, fluorescence quenching of RF with excess dsDNA was performed. RF fluorescence quenching was observed (Figure 3), indicating RF binding with dsDNA. Next, the CD spectroscopy of the dsDNA with or without RF was analyzed to determine the influence of RF on DNA structure. It was found that the presence of RF did not alter the structure of the dsDNA (Figure 2). Thus, RF binds DNA in a manner that does not affect the structure of dsDNA.

### 2.2. Identification of Guanine Oxidation Products in Double-Stranded DNA

**Figure 4:**
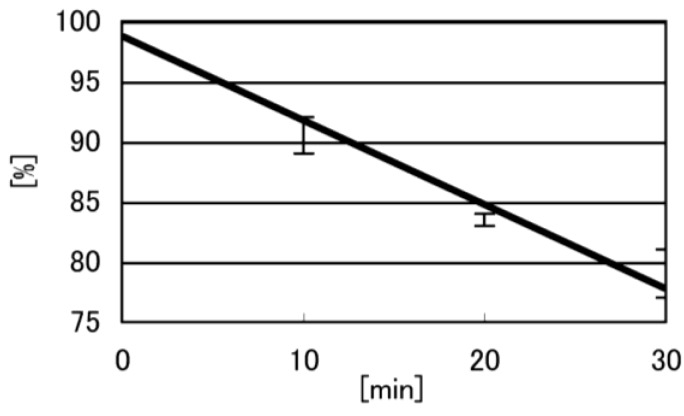
Time course of guanine oxidation by UVA in double-stranded DNA. dsDNA (700 µM) with 75 µM RF in 10 mM KCl, 5 mM cacodylate buffer (pH 7) was irradiated at 365 nm for 10, 20, and 30 min. The amount of d(TGGGGT) was determined using HPLC and absorbance monitored at 260 nm. The Y-axis represents the ratio of undamaged DNA to all DNA.

**Figure 5:**
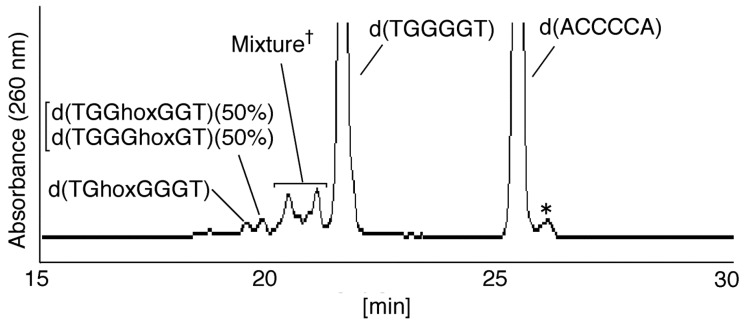
HPLC analysis of photooxidation products in double-stranded DNA. dsDNA (700 µM) with 75 µM RF in 10 mM KCl was irradiated at 365 nm for 30 min, and the guanine oxidation products were analyzed using HPLC (linear gradient of 0%–10% CH_3_CN/30 min) and absorbance monitored at 260 nm. “*” indicatesthe products which were not formed from d(TGGGGT). HPLC analysis of the mixture (†) was performed using a gradient HPLC with isocratic elution using 4% CH_3_CN/30 min (see section 2.2.2).

In a 10 mM KCl solution, dsDNA (700 µM) was photooxidized with 75 µM RF, and the products were analyzed using HPLC. To identify the oxidation products at low conversion rates (approximately 20%) [17,23], we first determined the rates of dsDNA oxidation. The oxidation rate of dsDNA is shown in Figure 4, and the conversion rates of the dsDNA at 30 min reached approximately 20%. Therefore, dsDNA was irradiated at 365 nm for 30 min and the guanine oxidation products were analyzed using HPLC (linear gradient of 0%–10% CH_3_CN/30 min); the HPLC profiles are shown in Figure 5.

#### 2.2.1. Isolation and Identification of Oligomers Containing Ghox

**Figure 6:**
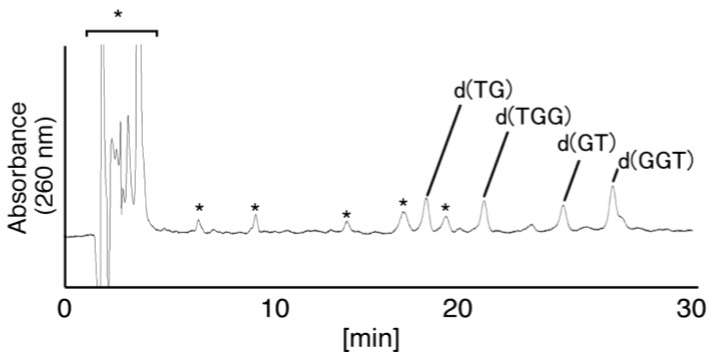
Piperidine treatment of oligomers containing Ghox. The products at 20.0 min in Figure 5 were isolated and heated with 1 M piperidine at 90 °C for 20 min, and subsequently dephosphorylated with alkaline phosphatase. The products compared with the commercial standard samples, dT, d(TG), d(TGG), d(TGGG), d(GT), d(GGT), and d(GGGT) using HPLC. Samples were analyzed using HPLC with a linear gradient of 3%–5% CH_3_CN/30 min and absorbance monitored at 260 nm. “*” indicates the products which were also detected in the negative control.

In Figure 5, four major peaks were detected at 19.6 and 20.0 min, with the peaks at 20.5 and 21.2 min having shoulder peaks. Two products (19.6 and 20.0 min) from Figure 5 were isolated and analyzed using electrospray ionization-mass spectrometry (ESI-MS), and the mass spectrum of an oligomer containing Ghox ([C_59_H_75_N_24_O_37_P_5_] *m/z* 932.16785, calculated for [M–2H] 932.16228) was identified (Figure 1) in both products. Four oligomers containing Ghox, d(TGhoxGGGT), d(TGGhoxGGT), d(TGGGhoxGT), and d(TGGGGhoxT) were synthesized as standards [17], and HPLC analysis of the standards was performed under the same HPLC gradient conditions as in Figure 5 (linear gradient of 0%–10% CH_3_CN/30 min). The retention times of d(TGhoxGGGT), d(TGGhoxGGT), d(TGGGhoxGT), and d(TGGGGhoxT) were identified as 19.6, 20.0, 20.0, and 18.8 min, respectively. Therefore, the peak at 19.6 min in Figure 5 was identified as d(TGhoxGGGT), and the peak at 20.0 min contained d(TGGhoxGGT) and/or d(TGGGhoxGT). The site of Ghox in the oligomer at 20.0 min in Figure 5 was identified subsequently by piperidine treatment (Figure 6), as previously reported [24,25]. As a result, the peak at 20.0 min in Figure 5 was found to contain d(TGGhoxGGT) (50%) and d(TGGGhoxGT) (50%). 

**Figure 7:**
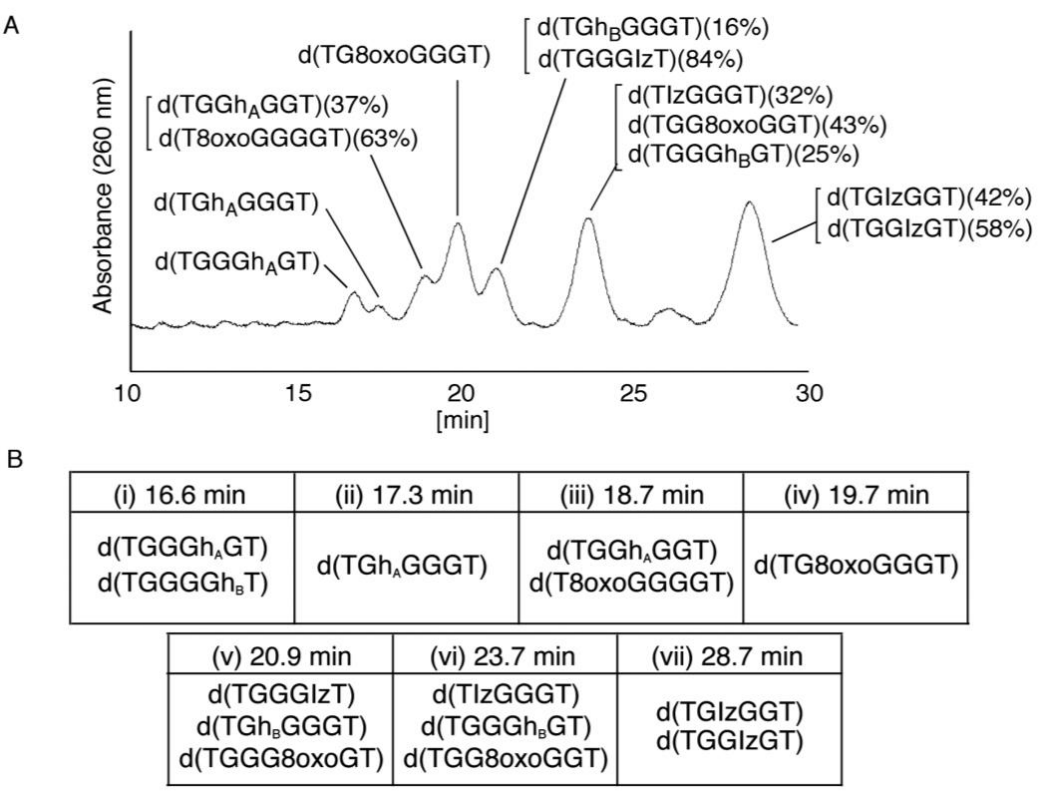
HPLC analysis of the mixed products. (**A**) The mixture in Figure 5 was isolated, and the products were analyzed using HPLC (isocratic flow using 4% CH_3_CN/30 min) and absorbance monitored at 260 nm; (**B**) The retention times of the standard samples were determined using HPLC (isocratic flow using 4% CH_3_CN/30 min).

**Figure 8:**
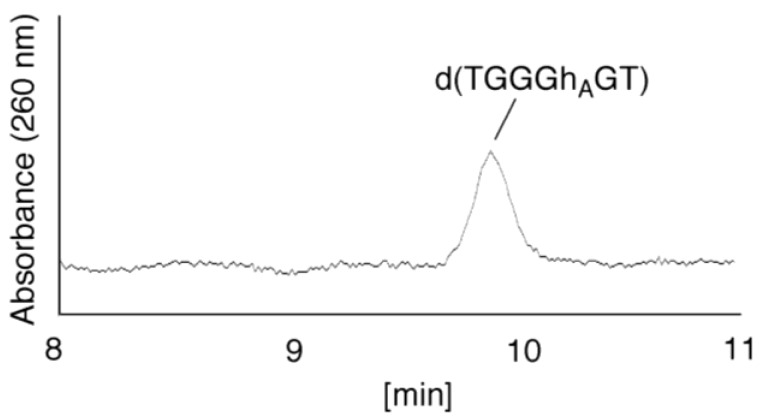
d(TGGGhGT) was detected using HPLC. The peak at 16.6 min in Figure 7A was isolated, and subjected to HPLC (linear gradient of 4%–10% CH_3_CN/30 min) and absorbance monitored at 260 nm.

**Figure 9:**
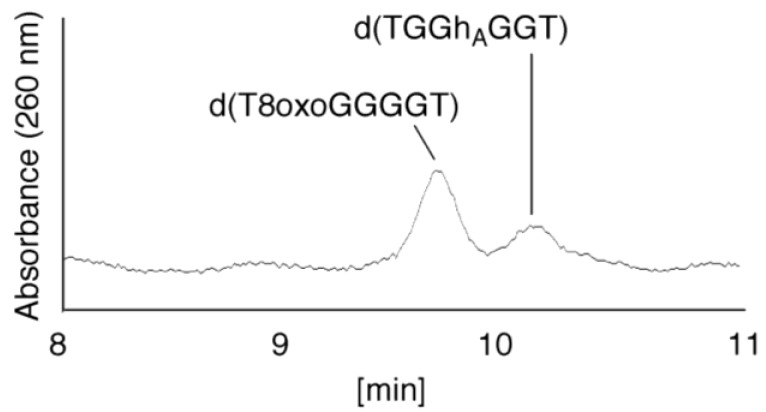
d(TGGhGGT) and d(T8oxoGGGGT) were detected using HPLC. The peak at 18.7 min in Figure 7A was isolated and analyzed using HPLC (linear gradient of 4%–10% CH_3_CN/30 min) and absorbance monitored at 260 nm.

**Figure 10:**
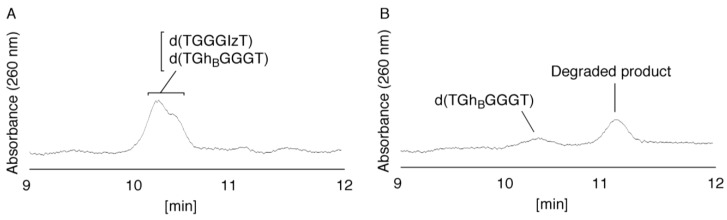
Analysis of the mixture containing d(TGGGIzT), d(TGhGGGT), and d(TGGG8oxoGT). The peak at 20.9 min in Figure 7A was isolated and heated to 80 °C for (**A**) 0 min or (**B**) 60 min. The samples were analyzed using HPLC with a linear gradient of 4%–10% CH_3_CN/30 min and absorbance monitored at 260 nm.

**Figure 11:**
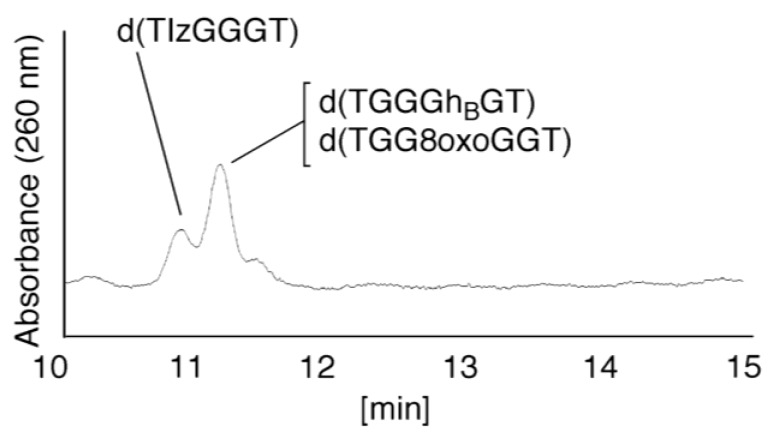
HPLC analysis of the mixture containing d(TIzGGGT), d(TGGGhGT), and d(TGG8oxoGGT). The peak at 23.7 min in Figure 7A was isolated and analyzed using HPLC (linear gradient of 4%–10% CH_3_CN/30 min) and absorbance at 260 nm monitored.

Next, the peaks at 20.5 and 21.2 min (Figure 5) were isolated and analyzed by ESI-MS, and a mixed mass spectrum of oligomers containing Iz ([C_58_H_74_N_23_O_36_P_5_] *m/z* 910.66027, calculated for [M–2H] 910.65937), guanidinohydantoin (Gh) ([C_59_H_77_N_24_O_37_P_5_] *m/z* 933.17775, calculated for [M–2H] 933.17010), and 8oxoG ([C_60_H_75_N_24_O_37_P_5_] *m/z* 938.16328, calculated for [M–2H] 938.16228) were detected. To separate the products in the mixture, HPLC analysis was performed using a modified HPLC gradient (isocratic flow using 4% CH_3_CN/30 min). The results are shown in Figure 7A and discussed in the next section. Additionally, due to the existence of two Gh diastereomers [26], two diastereomers of the oligomer containing Gh can be separated using HPLC [26]. In this study, Gh_A_ was defined as the Gh with a shorter retention time, and Gh_B_ with the longer retention time. This nomenclature is used in Figure 7, Figure 8, Figure 9, Figure 10 and Figure 11.

#### 2.2.2. Isolation and Identification of Oligomers Containing Iz, Gh or 8oxoG

In Figure 7A, seven major peaks were detected at 16.6, 17.3, 18.7, 19.7, 20.9, 23.7, and 28.7 min. The oligomers containing guanine oxidation products, d(TXGGGT), d(TGXGGT), d(TGGXGT) or d(TGGGXT) (X = Iz, Gh or 8oxoG) were synthesized and used as standard samples. The retention times of the standards (isocratic flow using 4% CH_3_CN/30 min) are shown in Figure 7B. The seven major peaks in Figure 7A were identified as follows.

**Figure 12:**
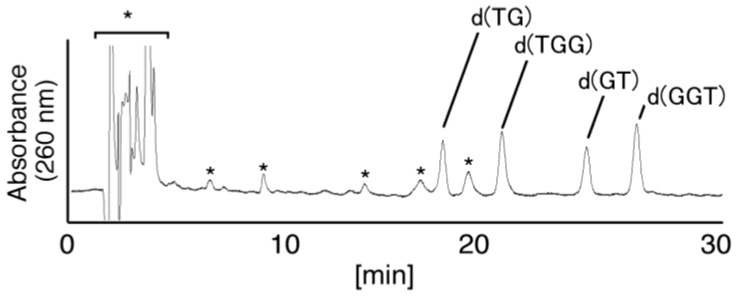
Piperidine treatment of oligomers containing Iz. The products in the 28.7 min peak in Figure 7A were isolated and heated with 1 M piperidine at 90 °C for 20 min, and subsequently dephosphorylated with alkaline phosphatase. The products were compared with the commercial standards dT, d(TG), d(TGG), d(TGGG), d(GT), d(GGT), and d(GGGT) using HPLC. Samples were analyzed using HPLC (linear gradient of 3%–5% CH_3_CN/30 min) and absorbance at 260 nm monitored. “*” indicates the products which were also detected in the negative control.

(i) Since the retention times of the standards d(TGGGhGT) and d(TGGGGhT) were identified as 16.6 min (Figure 7B), it was surmised that the peak at 16.6 min in Figure 7A contains d(TGGGhGT) and/or d(TGGGGhT). To distinguish d(TGGGhGT) from d(TGGGGhT), HPLC analysis using modified conditions (linear gradient of 4%–10% CH_3_CN/30 min in Figure 8) was performed, and the retention times of the standard samples d(TGGGGhT) and d(TGGGhGT) were identified as 9.3 and 9.7 min (data not shown). The 16.6 min peak in Figure 7A was isolated and reanalyzed using the HPLC conditions of Figure 8, which revealed that the 16.6 min peak in Figure 7A produced a single peak at 9.7 min. Thus, the peak at 16.6 min in Figure 7A contains only d(TGGGhGT).

(ii) Since the retention time for the standard d(TGhGGGT) (isocratic flow using 4% CH_3_CN/30 min) was 17.3 min (Figure 7B), the peak at 17.3 min in Figure 7A was identified as d(TGhGGGT).
(iii) Since the retention times of the standards d(T8oxoGGGGT) and d(TGGhGGT) (isocratic flow using 4% CH_3_CN/30 min) were both found to be 18.7 min (Figure 7B), the peak at 18.7 min in Figure 7A contains d(T8oxoGGGGT) and/or d(TGGhGGT). To distinguish d(T8oxoGGGGT) and d(TGGhGGT), modified HPLC conditions (linear gradient of 4%–10% CH_3_CN/30 min in Figure 9) were used to determine the retention times of the standards d(T8oxoGGGGT) and d(TGGhGGT), which were found to be 9.8 and 10.2 min, respectively. The peak at 18.7 min in Figure 7A was isolated and reanalyzed using HPLC (linear gradient of 4%–10% CH_3_CN/30 min) (Figure 9) and two peaks were detected at 9.8 and 10.2 min. The ratio of the peak areas at 9.8 and 10.2 min in Figure 9 was determined as 63:37. Thus, the peak at 18.7 min in Figure 7A contains d(T8oxoGGGGT) (63%) and d(TGGhGGT) (37%).

(iv) Since the retention times of the standard d(TG8oxoGGGT) (a isocratic flow using 4% CH_3_CN/30 min) was identified as 19.7 min (Figure 7B), the peak at 19.7 min in Figure 7A was identified as d(TG8oxoGGGT).
(v) Since the retention times of the standards d(TGGGIzT), d(TGhGGGT), and d(TGGG8oxoGT) (isocratic flow using 4% CH_3_CN/30 min) were all 20.9 min (Figure 7B), the peak at 20.9 min in Figure 7A was presumed to contain d(TGGGIzT), d(TGhGGGT), and/or d(TGGG8oxoGT). Unfortunately, HPLC conditions that enabled separation of these three products were not found. Since HPLC with electrochemical detection (HPLC-ECD) can specifically detect oligomers containing 8oxoG, the peak at 20.9 min in Figure 7A was analyzed using this method. However, oligomers containing 8oxoG were not detected.
  Iz is degraded to 2,2,4-triamino-5(2*H*)-oxazolone (Oz) [27,28,29], while Gh and 8oxoG are the stable products [30,31]. A standard oligomer containing Iz was hardly detected in a solution heated to 80 °C for 60 min. Therefore, d(TGGGIzT) in the mixture was degraded and the amount of d(TGGGIzT) could be determined by measuring peak area loss. The peak at 20.9 min in Figure 7A was isolated and heated to 80 °C for 60 min. This resulted in the peak area decreasing to 16% of the original (Figure 10). Hence, the peak at 20.9 min in Figure 7A was determined to be d(TGhGGGT) (16%) and d(TGGGIzT) (84%).

(vi) Since the retention times of the standards d(TIzGGGT), d(TGGGhGT), and d(TGG8oxoGGT) (isocratic flow using 4% CH_3_CN/30 min) were identified as 23.7 min (Figure 7B), it was surmised that the peak at 23.7 min in Figure 7A contains d(TIzGGGT), d(TGGGhGT), and/or d(TGG8oxoGGT). Therefore, HPLC analysis using modified HPLC conditions (linear gradient of 4%–10% of CH_3_CN/30 min in Figure 11) was performed to separate the three products. The retention times of d(TIzGGGT), d(TGGGhGT), and d(TGG8oxoGGT) were identified as 10.9, 11.2, and 11.2 min under these conditions. The peak at 23.7 min in Figure 7A was isolated and reanalyzed using HPLC with a linear gradient of 4%–10% CH_3_CN/30 min (Figure 11). This resulted in peaks being detected at 10.9 and 11.2 min and the ratio of these peak areas were determined to be 32:68, respectively. Therefore, d(TIzGGGT) accounts for 32% of the peak area at 23.7 min in Figure 7A.
  Unfortunately, d(TGGGhGT) and d(TGG8oxoGGT) could not be separated using HPLC. The peak at 23.7 min in Figure 7A was then analyzed using HPLC-ECD. This analysis revealed that the oligomer containing 8oxoG accounts for 43% of the peak area at 23.7 min in Figure 7A. Hence, it was determined that the peak at 23.7 min in Figure 7A contained d(TIzGGGT) (32%), d(TGGGhGT) (25%), and d(TGG8oxoGGT) (43%).

(vii) Since the retention times of the standards d(TGIzGGT) and d(TGGIzGT) (isocratic flow using 4% CH_3_CN/30 min) were both 28.7 min (Figure 7B), the peak at 28.7 min in Figure 7A contains d(TGIzGGT) and/or d(TGGIzGT). Therefore, the location of Iz in the oligomer at 28.7 min in Figure 7A was identified by piperidine treatment, as previously reported (Figure 12) [25,32]. As a result, the peak at 28.7 min in Figure 7A was identified as containing d(TGIzGGT) (42%) and d(TGGIzGT) (58%).

**Figure 13:**
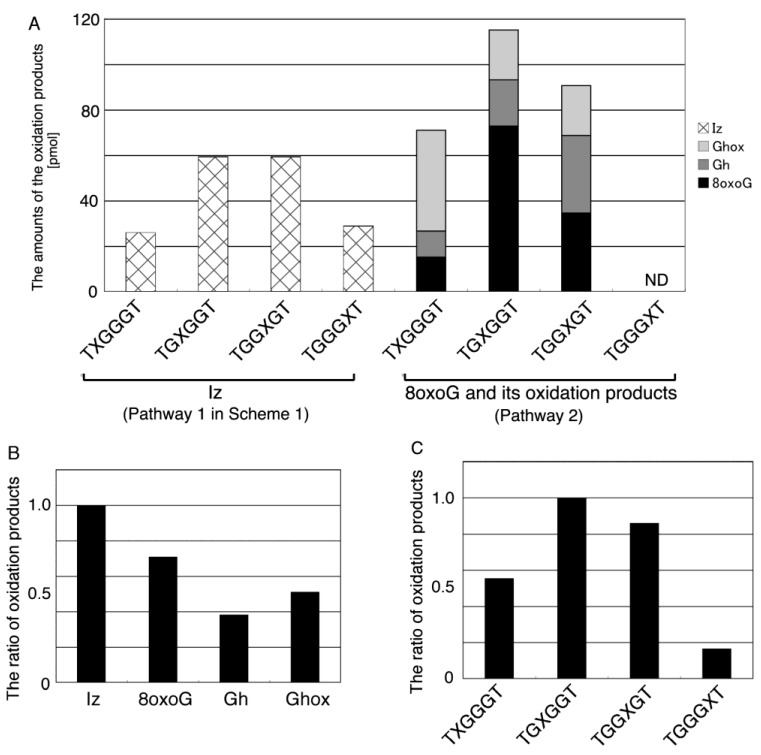
The types and site reactivities of guanine photooxidation products in double-stranded DNA. (**A**) The amounts of oxidation product. Iz is formed via Pathway 1 in Scheme 1, and 8oxoG, Gh, and Ghox are formed via Pathway 2 in Scheme 1 (see Section 2.3). The oligomers containing 8oxoG and its oxidation products are shown on the right half of panel A, and the oligomers containing Iz are shown on the left half. TGGG8oxoGT, TGGGGhT, and TGGGGhoxT were not detected (ND); (**B**) The proportions of oxidation product types; (**C**) The site of reactivity of the oxidation products.

Thus, we identified the types, yields and sites of the guanine oxidation products in dsDNA. The oxidation products in dsDNA were identified as Iz, 8oxoG, Ghox, and Gh, and their amounts are shown in Figure 13A. TG8oxoGGGT was determined as the main product, and the ratios of TGGIzGT and TGIzGGT to TG8oxoGGGT were 0.81. The total proportions of each product were calculated and presented in Figure 13B. Iz was the main product formed, and the ratios of 8oxoG, Ghox, and Gh to Iz were determined to be 0.71, 0.53, and 0.39, respectively. 

The total proportions of d(TIzGGGT), d(T8oxoGGGGT), d(TGhoxGGGT), and d(TGhGGGT) were calculated as TXGGGT in Figure 13C. Furthermore, TGXGGT, TGGXGT, and TGGGXT in Figure 13C were similarly calculated. As a result, the second guanine from the 5’ end was mainly oxidized in dsDNA, and the ratios of TGGXGT, TXGGGT, and TGGGXT to TGXGGT were determined to be 0.87, 0.58, and 0.16, respectively. 

Herein, dsDNA was prepared in 10 mM KCl, so that the photooxidation of dsDNA might be influenced by the coexistence of quadruplex DNA. However, the peak of the quadruplex DNA was not found in Figure 5 as the previous report [17]. Further, both d(TGGG8oxoGT) and d(TGGGGhoxT), which were mainly formed in the oxidation of quadruplex DNA [17], were also not detected in Figure 13A. Thus, the oxidation of ds DNA was not involved in quadruplex DNA.

**Scheme 1:**
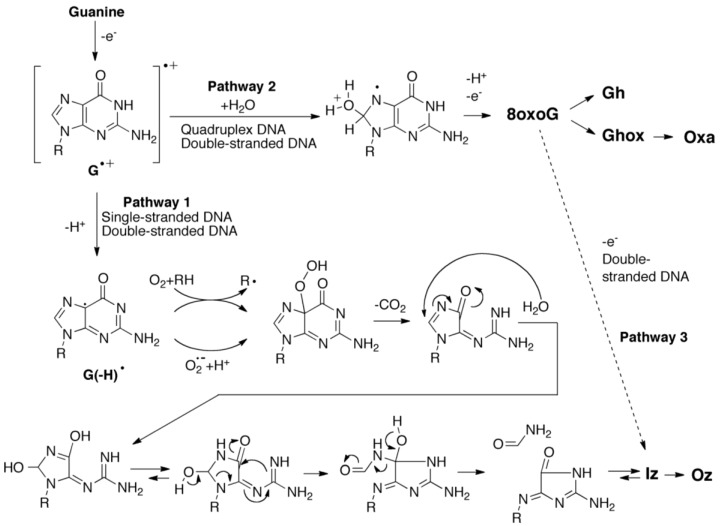
Proposed guanine oxidation pathways in single-stranded, double-stranded or quadruplex DNA.

### 2.3. Proposed Guanine Oxidation Pathways

It is said that one-electron oxidation [27] and oxidation with singlet oxygen [33,34] are mediated by RF under UVA irradiation [35]. To determine whether singlet oxygen was involved in the oxidation of guanine, dsDNA was photooxidized in 80% D_2_O, and the amounts of these oxidation products were not increased in the presence of 80% D_2_O (data not shown). Thus, the guanine oxidations in dsDNA undergo one-electron oxidation. We previously confirmed that guanine oxidation in single-stranded or quadruplex DNA also undergo one-electron oxidation [17]. To explain the differences between the one-electron oxidation products in single-stranded, double-stranded and quadruplex DNA, proposed mechanisms of guanine oxidation are discussed as follows.

**Scheme 2:**
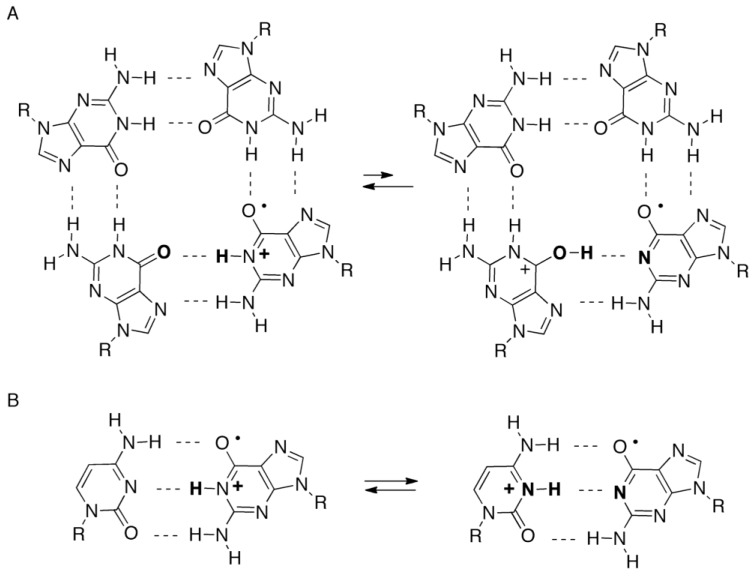
Proton shifts in quadruplex and double-stranded DNA. (**A**) The proton shift in G^•+^/G base pair in quadruplex DNA; (**B**) The proton shift in G^•+^/C base pair in dsDNA.

We previously reported that the major photooxidation products of guanine in quadruplex DNA were 8oxoG and its oxidation product Ghox, while the major photooxidation product in single-stranded DNA was Iz [17]. A mechanism regulating the oxidation products in single-stranded or quadruplex DNA was proposed (Scheme 1) [17,36]. In these proposed pathways, one-electron oxidation of guanine generates a guanine radical cation (G^•+^) [37], and G^•+^ is subsequently modified in two competitive pathways. In single-stranded DNA, G^•+^ is deprotonated at the N1 position and the neutral guanine radical [G(-H)^•^] is formed (pathway 1). Thereafter, the formation of peroxide [38,39] and the nucleophilic addition of water induce a subsequent rearrangement [40], leading to the formation of Iz. The hydrogen bond between the N1 proton of G^•+^ and the O6 of its neighbor guanine forms in quadruplex DNA (Scheme 2A), and the hydrogen bond in quadruplex DNA is more strongly retained than in single-stranded DNA [17]. Therefore, deprotonation at the N1 position of G^•+^ (pathway 1) can be blocked in quadruplex DNA and hydration of G^•+^ results (pathway 2). In this case, the 8-hydroxy-7,8-dihydroguanyl radical is formed [41] and subsequently oxidized to 8oxoG in quadruplex DNA. Hence, the type of the guanine oxidation products may depend on the ease of deprotonation at the N1 position of G^•+^. 

In dsDNA, the hydrogen bond between the N1 proton of G^•+^ and the N3 of base-paired cytosine is formed (Scheme 2B). The oxidation products in dsDNA are also likely to depend on the ease of deprotonation at the N1 position of G^•+^ in dsDNA. Therefore, the ease of deprotonation in single-stranded, double-stranded, or quadruplex DNA is discussed below. 

The deprotonation of G^•+^ in single-stranded, double-stranded and quadruplex DNA is represented as Equations (1)–(3). The equilibrium constants of the reactions in Equations (1)–(3) are defined as *K_s_, K_d_*, and *K_q_*, respectively. *K_s_*, *K_d_*, and *K_q_* are defined by Equations (4)–(6). To determine the deprotonation ratios, *K_d_/K_s_* is obtained according to Equation (7), which is derived from Equations (4) and (5). *K_q_/K_s_* is obtained according to Equation (8), which is derived from Equations (4) and (6).

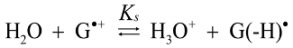
(1)

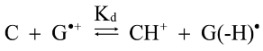
(2)

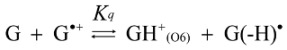
(3)

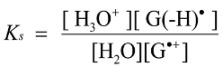
(4)

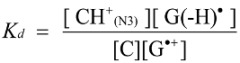
(5)

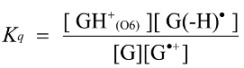
(6)

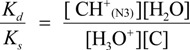
(7)

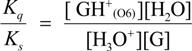
(8)


The acid dissociation constants for the N3 protonated cytosine (Equation (2)) and the O6 protonated guanine (Equation (3)) are defined as *K_a_*_(N3)_ and *K_a_*_(O6)_ in Equations (9) and (10), with the dissociation constants obtained using Equations (11) and (12). Thus, *K_d_/K_s_* and *K_q_/K_s_* are obtained using Equations (13) and (14), which are derived from Equations (7), (8), (11), and (12).

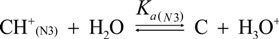
(9)

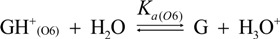
(10)

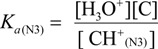
(11)

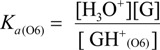
(12)

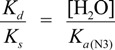
(13)

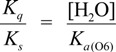
(14)


Therefore, *K_a_*_(N3)_, *K_a_*_(O6)_, and [H_2_O] are necessary to determine *K_d_/K_s_* and *K_q_/K_s_*. The concentration of water is 55.6 M, and *K_a_*_(N3)_ is 2.5 × 10^4^ [42]. *K_a_*_(O6)_ itself is not reported, but *K_a_*_(O6)_ can be derived from the pK_a_ of the N7 protonated guanine (p*K_a_*_(N7)_) [42] and the relative free energy of the O6 protonated guanine with respect to the N7 protonated guanine in the aqueous phase (*ΔG*_(N7-O6)_) [42], so that *K_a_*_(O6)_ can be calculated. As a result, *K_a_*_(O6)_ is determined to be 3.2 × 10^5^. Therefore, *K_d_/K_s_* is found to be 2.2 × 10^−3^, and *K_q_/K_s_* is 1.7 × 10^−4^. Thus, *K_d_* is smaller than *K_s_*, and larger than *K_q_*. 

Thus, we demonstrated that deprotonation in dsDNA is easier than in quadruplex DNA and is more difficult than in single-stranded DNA. This discussion of the deprotonation equilibrium constant supports the contention that both reactions in pathways 1 and 2 (Scheme 1) are likely to occur in dsDNA. This mechanism explains why the major oxidation products in dsDNA were consistent with a combination of each major oxidation product in single-stranded and quadruplex DNA.

Additionally, Iz and 8oxoG were also formed following the oxidation of double-stranded d(TTGGTA)/d(AAACCATA) [25] or d(A_6_GGA_6_)/d(T_6_CCT_6_) [43]. Iz was mainly formed by the oxidation of double-stranded d(TTGGTA)/d(AAACCATA) [25], and the amount of 8oxoG was significantly less than that of Iz. In contrast, 8oxoG was mainly formed by the oxidation of double-stranded d(A_6_GGA_6_)/d(T_6_CCT_6_) [43] at a 20% conversion rate. Since the number of guanines in d(TTGGTA)/d(AAACCATA) is less than in d(TGGGGT)/d(ACCCCA), the double-stranded structure of d(TTGGTA)/d(AAACCATA) is more weakly retained and deprotonation at the N1 position of G^•+^ in d(TTGGTA)/d(AAACCATA) is also easier. The features of deprotonation support that the reactions in pathway 1 (Scheme 1) mainly occur in d(TTGGTA)/d(AAACCATA). Similarly, the number of base pairs in d(A_6_GGA_6_)/d(T_6_CCT_6_) is significantly larger than in d(TGGGGT)/d(ACCCCA), so that the deprotonation at the N1 position of G^•+^ in d(A_6_GGA_6_)/d(T_6_CCT_6_) is also more difficult. The features of deprotonation support the reactions in pathway 2 (Scheme 1) occurring mainly in d(A_6_GGA_6_)/d(T_6_CCT_6_). Thus, the ratio of 8oxoG to Iz depends on the stability of the double-stranded structure, and stabilization of double-stranded structures increase the ratio of 8oxoG. 

### 2.4. Formation of Iz by One-Electron Oxidation of Double-Stranded DNA

Following one-electron oxidation of d(A_6_GGA_6_)/d(T_6_CCT_6_), 8oxoG is further oxidized to Iz [43], and Iz is subsequently degraded to Oz [27,28,29] (Scheme 1). Oz is thought to be a factor in G-C transversion mutations, and studies of Oz have been previously reported [28,44,45]. The previous section describes the generation of 8oxoG in stable dsDNA, and Oz is ultimately formed via one-electron oxidation and the degradation of 8oxoG (pathway 3 in Scheme 1). Therefore, G-C transversion mutations are likely to be induced following the one-electron oxidation of dsDNA.

### 2.5. Formation of 8oxoG in Quadruplex DNA and Its Effects

In oxidized quadruplex DNA, a quadruplex DNA structure containing three d(TGGGGT) and one d(TGGG8oxoGT) might be formed. We attempted to experimentally determine whether quadruplex DNA containing d(TGGG8oxoGT) was formed. Therefore, d(TGGGGT) (210 µM) and d(TGGG8oxoGT) (70 µM) in 10 mM KCl were heated to 80 °C for 5 min and incubated at 4 °C for 1 min, and the solution was analyzed using ESI-MS. The resulting quadruplex DNA containing three d(TGGGGT) and one d(TGGG8oxoGT) was not detected, while quadruplex d(TGGGGT)_4_ and quadruplex d(TGGG8oxoGT)_4_ were detected (data not shown). Thus, the 8oxoG was formed in quadruplex DNA, and the quadruplex DNA subsequently dissociated to four single-stranded DNA molecules. Indeed, the generation of 8oxoG in quadruplex DNA has been observed to result in the formation of undamaged single-stranded DNA [17]. Single-stranded oligomers containing 8oxoG were observed to be further oxidized to Ghox instead of Iz [17]. Ghox is degraded to oxaluric acid (Oxa) [46] (Scheme 1), and Oxa is considered to be a factor in G-T transversion mutations [47]. Meanwhile, the oxidation of quadruplex DNA at high conversion rates is thought to result in the formation of oligomers containing Iz since undamaged single-stranded DNA is formed in the oxidation of quadruplex DNA [17]. Hence, under high oxidation conditions, Iz can also be formed in quadruplex DNA.

### 2.6. Localization of Guanine Oxidation Products

**Figure 14:**
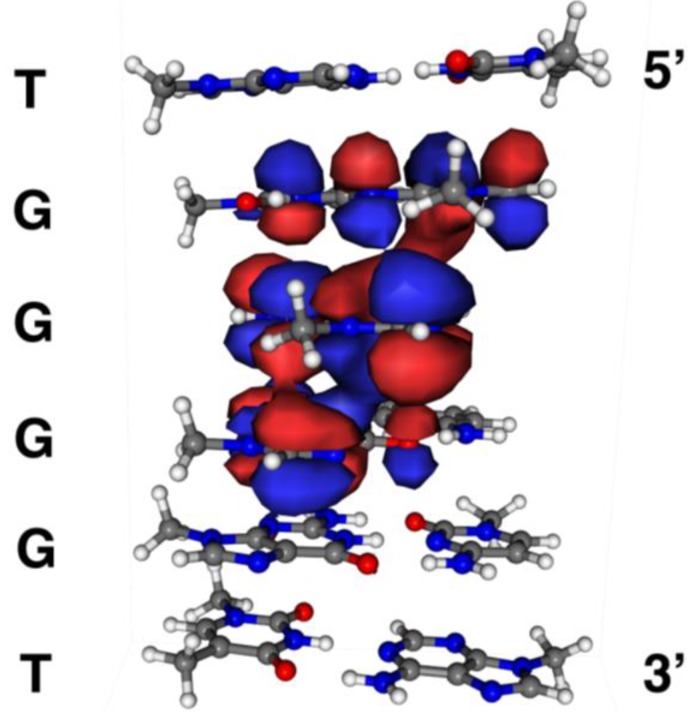
HOMO localization sites in double-stranded DNA.

The specific one-electron oxidation of guanine in contiguous guanine sequences, such as GG and GGG, in dsDNA was reported previously, and is dependent on the localization of the highest occupied molecular orbital (HOMO) [48,49,50]. Additionally, specific oxidation in quadruplex DNA also depends on the localization of HOMO [17]. Herein, the site reactivity in double-stranded d(TGGGGT)/d(ACCCCA) is likely to depend on the localization of HOMO. Therefore, we calculated the localization of HOMO in dsDNA. The calculation estimated that HOMO was mainly localized on the second guanine from the 5’ end (Figure 14). In addition, the third guanine from the 5’ end was a second HOMO localization site, and the first guanine from the 5’ end was a third HOMO localization site. Furthermore, the localization of HOMO at the 3’ end was rarely seen. In the generation of 8oxoG and its oxidation products, the second guanine from the 5’ end was primarily oxidized (Figure 13A), with the experimental result completely matching the calculations. Additionally, the agreement between the calculated and experimental results indicates that the one-electron oxidation of guanine occurred in dsDNA, and is in agreement with the experiments using D_2_O.

Moreover, the yield of TGIzGGT was greater than that of TIzGGGT, and the yield of TGGIzGT was greater than TGGGIzT (Figure 13A). Thus, the experimental studies corresponded well with the calculations (Figure 14). However, the yield of TGIzGGT was equal to that of TGGIzGT in the experimental studies (Figure 13A), and the experimental results did not match the calculated results. Furthermore, TGGGIzT was detected in experimental studies (Figure 13A), while HOMO on the 3’ end was not observed (Figure 14). Iz was also formed via oxidation of guanine in single-stranded DNA, and any of the four guanines was oxidized in single-stranded DNA. Since undamaged single-stranded DNA was also oxidized, the sites of Iz were not considered to be completely dependent on the localization of HOMO. The proposed pathway explains that the experimental result for the generation of Iz does not completely match the calculations in Figure 14. 

## 3. Experimental Section

### 3.1. Materials

Materials used for the experiments were purchased from the sources indicated below. P1 Nuclease was purchased from Takara Bio Inc. (Otsu, Japan). Alkaline phosphatase and reaction buffer were purchased from Toyobo Co., Ltd. (Osaka, Japan) CH_3_CN was purchased from Kanto Chemical Co., Inc. (Tokyo, Japan). AcONH_4_, cacodylic acid, dG and KCl were purchased from Wako Pure Chemical Industries, Ltd. (Osaka, Japan) Ammonium formate, NaOH, and piperidine were purchased from Nacalai Tesque Inc. (Kyoto, Japan) D_2_O was purchased from Merck Ltd. (Tokyo, Japan) Undamaged oligomers were purchased from Japan Bio Services Co., Ltd. (Asaka, Japan)

Oligonucleotides containing 8oxoG were synthesized using a conventional phosphoramidite method with a Polygen DNA synthesizer and commercially available phosphoramidites (Sigma-Aldrich, Tokyo, Japan). Following a previously described method, oligonucleotides containing Gh were produced from oligomers containing 8oxoG [29], and oligonucleotides containing Iz were produced from d(TGGGGT) [17]. Then, oligonucleotides containing Ghox were produced from oligomers containing 8oxoG [17].

### 3.2. Analysis of Oxidation Reactions

d(TGGGGT) and d(ACCCCA) in 10 mM KCl were heated to 80 °C for 5 min, and incubated at 4 °C for 1 min to form dsDNA. The CD of the dsDNA was determined using Jasco J-820 Circular Dichroism Spectropolarimeter. Fluorescence quenching of 6.25 µM RF by dsDNA (0–2.5 mM) in 10 mM KCl and 5 mM cacodylate buffer (pH 7) was performed using a Molecular Devices SpectraMax M5 plate reader. Photooxidation of dsDNA (700 µM) was conducted with 75 µM RF in 0.1 mM KCl irradiated at 365 nm using a UVP 3UV transilluminator. The oxidation products were analyzed by HPLC with a CHEMCOBOND 5-ODS-H column (Chemcopak, 5 μm, 150 × 4.6 mm) with a flow rate of 1.0 mL/min using two solvents, A and B, and absorbance at 260 nm monitored. Solvent A was 50 mM AcONH_4_ (pH 7) and solvent B was a CH_3_CN, and the column was equilibrated with solvent A. The gradient elution parameters were described in the figure legends. To determine the site of Ghox, Gh, or Iz, the oxidation products were heated with 1 M piperidine at 90 °C for 20 min and stored with alkaline phosphatase and reaction buffer at 37 °C for 3 h. The sample was then analyzed using HPLC. Further, oligomers containing 8oxoG were stored with P1 nuclease, alkaline phosphatase and reaction buffer at 37 °C for 2 h, and the digested samples were analyzed using HPLC with an electrochemical detector (Coulochem III, ESA). 

### 3.3. Calculation of the Highest Occupied Molecular Orbital (HOMO)

We calculated the localization of the HOMO, as previously reported [48,49,50]. The geometry of the stacked bases was constructed using the program Maestro 9.0 (Schrödinger) [51]. All the sugar backbones were removed, except for the 2’-deoxyribose C1 carbon and the C1 proton. Two H atoms were then attached to C1 to complete the N-methylated nucleobases. The calculation of HOMO was performed at the B3LYP/6-31G* level utilizing Gaussian 03 [52] and the localization of HOMO was visualized using the program Molekel 5.4.0.8 [53]. C2 of the second guanine from the 5’ end had the largest atomic charge with hydrogens summed into heavy atoms, and the 2px orbital had the largest molecular orbital coefficient. Using the molecular orbital coefficients for the 2px orbital of each guanine, the ratios of TGGXGT, TXGGGT, and TGGGXT to TGXGGT were determined to be 0.53, 0.45, and 0.10, respectively.

## 4. Conclusions

Identification of the guanine oxidation products in dsDNA was accomplished using HPLC and ESI-MS (Figure 5 and Figure 7). As a result, both major oxidation products found in single-stranded and quadruplex DNA were detected in dsDNA. In a previous report [17], the oxidation products in single-stranded or quadruplex DNA were found to depend on the ease of deprotonation at the N1 proton of G^•+^. We revealed that deprotonation in dsDNA is easier than in quadruplex DNA and is more difficult than in single-stranded DNA. The discussion herein explains that the major oxidation products in dsDNA are Iz, 8oxoG, Ghox and Gh. Furthermore, the second guanine from the 5’ end was mainly oxidized in dsDNA (Figure 13C), and the localization of HOMO was mainly localized on the second guanine from the 5’ end (Figure 14). Therefore, the specific oxidation in dsDNA depends on the HOMO localization in DNA structures.
